# Global transcriptomic analysis of an engineered *Escherichia coli* strain lacking the phosphoenolpyruvate: carbohydrate phosphotransferase system during shikimic acid production in rich culture medium

**DOI:** 10.1186/1475-2859-13-28

**Published:** 2014-02-21

**Authors:** Larisa Cortés-Tolalpa, Rosa María Gutiérrez-Ríos, Luz María Martínez, Ramón de Anda, Guillermo Gosset, Francisco Bolívar, Adelfo Escalante

**Affiliations:** 1 Departamento de Ingeniería Celular y Biocatálisis, Instituto de Biotecnología, Universidad Nacional Autónoma de México, Av. Universidad 2001, Col. Chamilpa, Cuernavaca, Morelos 62210, México; 2 Departamento de Microbiología Molecular, Instituto de Biotecnología, Universidad Nacional Autónoma de México, Av. Universidad 2001, Col. Chamilpa, Cuernavaca, Morelos 62210, México

**Keywords:** Shikimic acid production, *Escherichia coli* PTS- strain, Batch-fermentor culture, Complex fermentation broth, Global transcriptomic analysis, Microarrays, Regulatory network

## Abstract

**Background:**

Efficient production of SA in *Escherichia coli* has been achieved by modifying key genes of the central carbon metabolism and SA pathway, resulting in overproducing strains grown in batch- or fed-batch-fermentor cultures using a complex broth including glucose and YE. In this study, we performed a GTA to identify those genes significantly upregulated in an engineered *E. coli* strain, PB12.SA22, in mid EXP (5 h), early STA (STA1, 9 h), and late STA (STA2, 44 h) phases, grown in complex fermentation broth in batch-fermentor cultures.

**Results:**

Growth of *E. coli* PB12.SA22 in complex fermentation broth for SA production resulted in an EXP growth during the first 9 h of cultivation depending of supernatant available aromatic amino acids provided by YE because, when tryptophan was totally consumed, cells entered into a second, low-growth phase (even in the presence of glucose) until 26 h of cultivation. At this point, glucose was completely consumed but SA production continued until the end of the fermentation (50 h) achieving the highest accumulation (7.63 g/L of SA). GTA between EXP/STA1, EXP/STA2 and STA1/STA2 comparisons showed no significant differences in the regulation of genes encoding enzymes of central carbon metabolism as in SA pathway, but those genes encoding enzymes involved in sugar, amino acid, nucleotide/nucleoside, iron and sulfur transport; amino acid catabolism and biosynthesis; nucleotide/nucleoside salvage; acid stress response and modification of IM and OM were upregulated between comparisons.

**Conclusions:**

GTA during SA production in batch-fermentor cultures of strain PB12.SA22 grown in complex fermentation broth during the EXP, STA1 and STA2 phases was studied. Significantly, upregulated genes during the EXP and STA1 phases were associated with transport, amino acid catabolism, biosynthesis, and nucleotide/nucleoside salvage. In STA2, upregulation of genes encoding transporters and enzymes involved in the synthesis and catabolism of Arg suggests that this amino acid could have a key role in the fuelling of carbon toward SA synthesis, whereas upregulation of genes involved in pH stress response, such as membrane modifications, suggests a possible response to environmental conditions imposed on the cell at the end of the fermentation.

## Background

**Figure 1:**
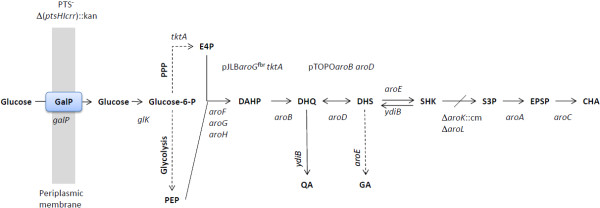
**Central carbon metabolism and shikimic acid pathways in evolved *E. coli *PB12.SA22 strain.** Glucose transport and phosphorylation are performed by galactose permease (GalP) and glucokinase (Glk), respectively. Genes and coded enzymes: *galP*, GalP; *glk,* Glk; *tktA,* transketolase I; *pykF*, pyruvate kinase I; *pykA*, pyruvate kinase II; *ppsA*, phosphoenolpyruvate synthase; *aroF, aroG, aroH*, DAHP synthase isoenzymes F, G and H, respectively; *aroB,* DHQ synthase; *aroD*, DHQ dehydratase; *aroE*, shikimate dehydrogenase*; aroK*, shikimate kinase I; *aroL*, shikimate kinase II; *aroA*, EPSP synthase; *aroC*, chorismate synthase. pJLB*aroG*^fbr^*tktA* and pTOPO*aroBaroD*, indicate cloned plasmids for SA overproduction in this strain [10]. Continuous arrows represent unique reactions catalyzed by one or more enzymes; dotted lines or arrows represent two or more enzymatic reactions or incomplete characterized reactions. Cross line in the reaction of SHK to S3P indicates interruption of the SA pathway.

The SA pathway is the common route leading to the biosynthesis of aromatic compounds in bacteria and in several eukaryotic organisms such as ascomycetes fungi, Apicomplexa, and plants [1,2]. In *Escherichia coli*, the first step in this pathway is the condensation of the CCM intermediates PEP and E4P, into DAHP by the DAHP synthase isoenzymes AroF, AroG, and AroH, which are encoded by the *aroF, aroG,* and *aroH* genes, respectively (Figure 1). The DHQ synthase, encoded by *aroB*, converts DAHP into DHQ. Subsequently, DHQ dehydratase, encoded by *aroD*, converts DHQ into DHS, and this compound is then transformed to SA by shikimate dehydrogenase, which is encoded by *aroE*. Shikimate kinases I and II, encoded by *aroL* and *aroK*, respectively, convert SA into S3P. Finally, S3P is converted to EPSP by 3-phosphoshikimate-1-carboxyvinyltransferase, which is encoded by *aroA*. The last step in the SA pathway is the synthesis of CHA by the CHA synthase enzyme, which is encoded by *aroC.* CHA is the common building block for the formation of aromatic amino acids and compounds such as quinone, menaquinone and enterobactin [3-5].

SA is a commercially important compound because it is considered to be an enantiomerically pure building block that is used as the precursor for the synthesis of numerous chemicals. Currently, SA has gained great importance as the starting compound for the chemical synthesis of OSP, the selective and potent inhibitor of the neuraminidase enzyme located on the surface of the influenza virus, known commercially as Tamiflu® and produced by Roche Pharmaceuticals [3-5]. OSP prevents the release of newly formed virus particles from influenza virus types A and B, avian influenza virus H5N1 and, recently, human influenza virus H1N1. Since 1999, Roche Pharmaceuticals increased the production of OSP to ensure a significant reservoir in several countries in anticipation of a possible pandemic influenza outbreak; however, it has been estimated that in this scenario, the production of the antiviral would be insufficient to cover the requirements of the world population [6,7], particularly in developing countries such as Mexico. The latest human influenza outbreak, which appeared in Mexico in 2009, showed that production of OSP is clearly insufficient to satisfy the demand for this antiviral in an emergency situation. Additionally, the main supply of SA for OSP production is currently derived from the seeds of Chinese star anise (*Illicium verum*). The supply of this source is susceptible to vagaries of the weather. The star anise plant takes around six-years from planting to bear fruit but remains productive for a long time; additionally, extraction and purification from its seeds results in low yields. Thus, alternative biotechnological strategies with engineered bacterial strains to produce SA have gained relevance [4,5].

Metabolically engineered *E. coli* strains for SA production include several genetic modifications in CCM such as the introduction of an additional plasmid-copy DAHPS AroF^fbr^, encoded by *aroF*^fbr^ or DAHPS AroG^fbr^ (*aroG*^fbr^), the *tktA* gene encoding transketolase I, and genes encoding enzymes from the SA pathway, including the single or double inactivation of genes *aroK* and *aroL*, and the introduction of an additional plasmid-copy of genes encoding limiting enzymes of the pathway such as *aroB* and *aroE,* resulting in an increased carbon flux from the CCM intermediates PEP and E4P to the SA pathway and accumulation of SA. The above-described genetic modifications in specific *E. coli* genetic backgrounds with additional modifications and grown under diverse culture conditions have resulted in the successful overproduction of SA with yields ranging from 0.08 to 0.42 mol SA/mol glucose [8-13] (Table 1).

**Table 1:** ***E. coli *** **SA engineered overproducing strains, growth conditions and SA yield**

**Strain/derivative**	**Relevant characteristics**	**Culture conditions**	**SA [g/L]**	**Yield (mol SA/mol glucose)**	**Reference**
SP1.1/pKD12.138	*serA*::*aroB* Δ*aroL* Δ*aroK* pSU18*aroF*^fbr^P^tac^*aroE serA tktA*	1-L fed-batch cultures, mineral broth with 25 g/L of glucose and 15 g/L of YE	52	0.18	[8]
SP1.1*pts*/pSC6.090B	PTS^−^*serA::aroB* Δ*aroL* Δ*aroK* PTS^−^ P^tac^*glf glk aroF*^fbr^*tktA* P^tac^*aroE serA, glf*^*1*^*glk*^2^	10-L fed-batch reactors, mineral broth with 25 g/L of glucose and 15 g/L of YE	71	0.27	[8]
W3110.shik1	Δ*aroL aroG*^fbr^*aroF*^fbr^*tnaA,* and plasmid overexpressed *aroK*	Chemostat cultures using mineral broth under glucose limiting conditions	NR	~0.08	[9]
PB12.SA22 (JM101 derivative)	PTS^−^*ΔaroL ΔaroK* pJLB*aroG*^fbr^*tktA* pTOPO *aroB aroE*	0.5-L batch reactors, mineral broth with 25 g/L of glucose and 15 g/L of YE	7	0.29	[10]
DHPYAAS-T7(DH5α- derivative)	Δ*ptsHIcrr* Δ*aroL* Δ*arok* Δ*ydiB* knock-in of T7-RNA-pol gene, pAOC-TGEFB *aroE aroB,* site-specific mutagenesis *glk tktA aroF*^fbr^	Fed-batch fermentation, modified M9 medium, with 25 g/L of glycerol and 25 g/L of YE	1.85	NR	[11]
AR36 (JM101 derivative)	PB12 *lacI*^*−*^*aroK*^*−*^*aroL*^*−*^*pykF*^*−*^ Trc/*aroB*^+^*tktA*^+^*aroG*^fbr+^*aroE*^+^*aroD*^+^*zwf*^+^	Batch fermentation, mineral broth with 100 g/L of glucose and 30 g/L of YE	43	0.42	[12]
*E. coli* SA116	Chromosomally evolved and cofactor metabolic engineered strain	Mineral broth with 10 g/L of glucose, 1 g/L of peptone, 1.24 g/L of proline	3.12	0.33	[13]

^1^Glucose facilitator (*glf*) and ^2^Glucokinase (*glk*) from *Zymomonas mobilis*; NR, Non-reported.

Previous characterization of SA production in strain PB12.SA22 in 0.5-L batch-fermentor cultures using complex fermentation broth including 25 g/L of glucose and 15 g/L of YE showed a characteristic two-phase growth behavior with an initially EXP growth with high *μ* while consuming ~ one-third of the initially added glucose and low level production of SA and other pathway intermediates. During the second growth phase, the *μ* decreased, and the culture entered the STA phase despite the presence of abundant residual glucose in the supernatant broth, whereas SHK pathway intermediate production increased, continuously reaching its maximum until the end of the fermentation (50 h). Interestingly, residual glucose was depleted from supernatant culture during the STA phase associated with SA production [10]. This growth, glucose consumption and SA production behavior suggest that during the EXP growth phase, strain PB12.SA uses YE components to support growth and, as consequence of the possible depletion of an essential nutrient component, cell ceases growth upon entering the STA phase, where residual glucose was channeled by this strain to produce SA and other pathway intermediates. These data suggest the presence of important genetic regulation and physiological differences during the EXP and STA phases.

GTA has been proven to be a powerful tool to study regulation of cellular metabolism in response to specific environmental conditions. To our knowledge, in relation to SA production in *E. coli*, GTA has been used to elucidate byproduct formation in the SA production strain W3110.shik1 under carbon and phosphate-limited (carbon-rich) chemostat conditions, suggesting that byproduct formation under carbon limitation is explained by the upregulation of a set of genes coupled to the SA pathway [14]. In this study, we report the GTA in strain PB12.SA22 during SA production in batch-fermentor cultures using complex fermentation broth including 25 g/L of glucose and 15 of g/L YE. GTA was determined in this strain in the mid EXP growth phase (5 h), at the beginning of the STA phase (STA1) (9 h) and in the late STA phase (STA2) (44 h), and the comparison of differentially upregulated genes was established during the EXP/STA1, EXP/STA2 and STA1/STA2 phases to correlate changes in the global expression profile and growth, glucose consumption and SA production profiles in this strain.

## Results

### Growth and SA pathway intermediate production

**Figure 2:**
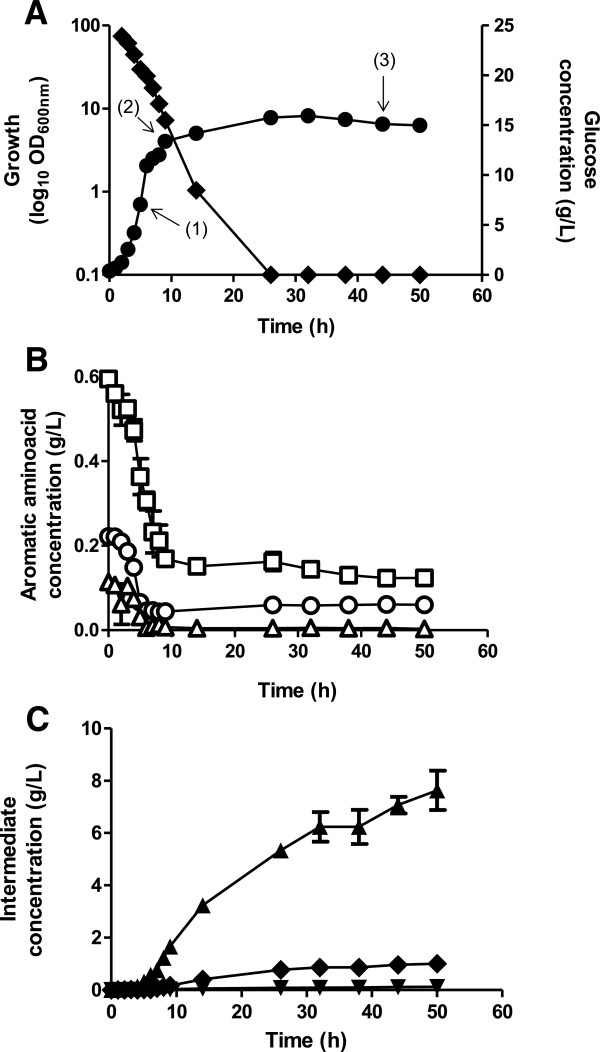
**Growth, aromatic intermediates production, and substrate consumption during batch fermentation cultures of *E. coli *PB12.SA22 strain grown in complex broth. (A)** Biomass production (●) Glucose consumption (◆). **(B)** Residual Phenylalanine (☐), Tyrosine (○), Tryptophan (△). **(C)** SA (▲), DHS (◆), GA (▼) production. In panel A, numbers in parenthesis indicate the sampling time of biomass for total RNA extraction used for microarray analysis (1), (2) and (3) indicates samples collected at 5, 9, and 44 h of cultivation, respectively.

Based on growth and glucose consumption profiles, strain PB12.SA22 showed its characteristic two-phase growth behavior [10], with an initial EXP growth phase during the first 8 h of cultivation reaching an OD_600 nm_ = 13.37 with a *μ* = 0.47 ± 0.002 h^−1^ and a *q*_*S*_ = 3.34 ± 0.29 mmol glucose g DW^−1^ h^−1^. After 8 h of cultivation, the culture reduced its growth rate even in the presence of ~70% of initially added glucose in the fermentation broth, and after 10 h of cultivation, the strain entered into a pseudo-STA phase in which a low growth phase was observed until 26 h of cultivation, where the highest OD_600 nm_ = 15.77 was observed. During this period, the strain completely consumed the residual glucose present in the supernatant broth (showing a *q*_*S*_ = 2.68 ± 0.5 mmol glucose g DW^−1^ h^−1^). From this point to the end of the fermentation, the biomass showed a slight reduction to OD_600 nm_ ~ 15 (Figure 2A). Interestingly, analysis of the consumption profile of available supernatant aromatic amino acids by HPLC showed that strain PB12.SA22 totally consumed the tryptophan present in the YE during EXP growth, reducing its growth when this aromatic amino acid was completely depleted from the culture supernatant (Figure 2B). SA production was detected in supernatant cultures in the EXP phase, but the highest SA accumulation was observed during the last 24 h, accumulating 7.63 g/L SA at the end of fermentation (50 h) (Figure 2C). The determined SA pathway byproducts, DAHP, DHS, and GA, appeared to be associated with growth because their production remained relatively constant after strain PB12.SA22 entered the STA phase. SA production resulted in a yield of 0.31 mol SA/mol glucose, and the total aromatic yield (the combined yield of SA, DAHP, DHS, and GA) was 0.36 mol aromatic intermediates/mol glucose.

### Global transcriptomic analysis during the EXP and STA phases

GTA analysis was determined by triplicate in the middle of the EXP phase (5 h), at the end of the EXP growth phase and upon entering pseudo-STA phase (9 h) (STA1) and at the end of the fermentation, or the late STA phase (44 h) (STA2). Average expression data were compared among EXP/STA1, EXP/STA2, and STA1/STA2, using the expression data of 4070 genes from the array of *E. coli* MG1655 included in the Affymetrix GeneChip® *E. coli* Genome 2.0 by the RP method. For all expression data, the RP method calculated a FDR. Those genes with an FDR value = 0 have the highest probability of biological relevance [15,16]. To identify differentially expressed (upregulated and downregulated) genes among compared growth conditions, an FDR value cutoff ≤ 0.05 was used. This analysis resulted in the identification of the significant upregulation of 43 genes and the downregulation of 80 genes in EXP/STA1, the upregulation of 37 genes and the downregulation of 38 genes in EXP/STA2, and the upregulation of 50 genes and the downregulation of 47 genes in ST1/STA2 (Table 2). Among them, 22 common genes were found to be upregulated during the entire fermentation process (EXP, STA1 and STA2). Biological functions were assigned to upregulated genes according to the EcoCyc database for *E. coli* strain MG1655 and broadly grouped on the basis of GO terms [17].

**Table 2:** **Differentially expressed genes during EXP/STA1, EXP/STA2 and STA1/STA2 comparisons during SA production**^**a**^

**GO terms**^**b**^	**EXP/STA1**		**EXP/STA2**		**STA1/STA2**	
	**UP**	**DR**	**UP**	**DR**	**UP**	**DR**
Transport	15	9	9	5	8	10
Amino acid metabolism	9	15	8	ND	16	4
Electron carrier activity	2	ND	1	ND	3	ND
Out of GOs	2	21	4	8	4	14
ATP catabolic/biosynthetic process	2	1	ND	ND	ND	ND
Nucleotide/nucleoside metabolism	6	2	1	ND	ND	2
Sulfur metabolism	2	ND	4	ND	2	ND
Stress response	1	12	2	6	5	3
Catalytic activity	1	ND	1	ND	ND	ND
Regulatory	2	7	4	7	4	4
Cell envelope	ND	5	2	7	1	4
Structural molecule activity	1	ND	1	ND	ND	ND
Lipid metabolism	ND	1	ND	ND	3	ND
Other metabolic process	ND	7	ND	5	4	6
Total gene number	43	80	37	38	50	47

^a^Based on resultant FDR value from the RP analysis equal or less to 0.05 [15]; ^b^ Biological functions were assigned according to the EcoCyc database for *E. coli* strain MG1655 and broadly grouped on the basis of GO terms [17]. ND, Non-detected.

Initial GTA data mining for upregulated genes in EXP/STA1 (Table 3), EXP/STA2 (Table 4), and STA1/STA2 (Table 5) comparisons resulted from the RP analysis from average transcription data for genes encoding enzymes involved in glycolysis, PPP, TCA, and the glyoxylate shunt, acetate metabolism and gluconeogenic capabilities in the EXP, STA1 and STA2 phases, showed that genes encoding proteins of the CCM and SA pathways were not significantly upregulated among performed comparisons (Additional file 1). However, significantly upregulated genes detected during the EXP and STA phases suggest important differences in sugar, amino acid, nucleotide/nucleoside and ion transport; metabolic process, particularly amino acid catabolism and biosynthesis; nucleotide/nucleoside salvage; acid stress response and modification of the IM. A detailed list of those differentially expressed genes in the EXP/STA1, EXP/STA2 and STA1/STA2 comparisons, expression data and FDR values information are presented in Additional file 1. The role and the relationship with growth, substrate consumption and SA production of relevant upregulated genes during EXP/STA1, EXP/STA2 and STA1/STA2 will be discussed below.

**Table 3:** Average expression data of upregulated genes during EXP/STA1 comparison based on resultant FDR value from the RP analysis equal or less to 0.05 in compared growth phases during SA production cultures

**Gene**	**Locus**	**Protein name**^**a**^	**Cellular function**^**b**^	**Average expression**
*asnA*	b3744	Asparagine synthetase A	Amino acid metabolism	28.5470
*aspA*	b4139	Aspartate ammonia-lyase	Amino acid metabolism	5.7446
*gcvH*	b2904	Glycine cleavage system H protein	Amino acid metabolism	7.2457
*gcvP*	b2903	Glycine decarboxylase	Amino acid metabolism	12.2509
*gcvT*	b2905	Aminomethyltransferase	Amino acid metabolism	12.0966
*putA*	b1014	Fused PutA DNA-binding transcriptional repressor/proline dehydrogenase/ 1-pyrroline-5-carboxylate dehydrogenase	Amino acid metabolism	11.8149
*sdaB*	b2797	L-serine deaminase II	Amino acid metabolism	8.2308
*tnaA*	b3708	L-cysteine desulfhydrase/tryptophanase	Amino acid metabolism	60.3464
*tnaL*	b3707	*tna* operon leader peptide	Amino acid metabolism	91.7140
*atpG*	b3733	ATP synthase F_1_ complex - gamma subunit	ATP biosynthetic/catabolic process	5.5487
*fecE*	b4287	Ferric dicitrate ABC transporter - ATP binding subunit	ATP biosynthetic/catabolic process	7.0184
*ompT*	b0565	OM protease VII (OM protein 3b)	Catalytic activity	6.0698
*groS*	B4142	Polypeptide: GroES, chaperone binds to Hsp60 in pres. Mg-ATP, suppressing its ATPase activity	Cell processes	5.9806
*cyoB*	b0431	Cytochrome *bo* terminal oxidase subunit I	Electron carrier activity	6.3920
*cyoD*	b0429	Cytochrome *bo* terminal oxidase subunit IV	Electron carrier activity	6.6316
*gpt*	b0238	Xanthine-guanine phosphoribosyltransferase	Nucleotide/nucleoside metabolism	6.4026
*guaC*	b0104	GMP reductase	Nucleotide/nucleoside metabolism	6.7694
*pyrB*	b4245	Aspartate carbamoyltransferase, catalytic subunit	Nucleotide/nucleoside metabolism	10.8487
*rihA*	b0651	Ribonucleoside hydrolase 1 (pyrimidine-specific)	Nucleotide/nucleoside metabolism	5.9568
*upp*	b2498	Uracil phosphoribosyltransferase	Nucleotide/nucleoside metabolism	5.4210
*yliF*	b0834	Predicted diguanylate cyclase	Nucleotide/nucleoside metabolism	5.6595
*nlpA*	b3661	Lipoprotein-28	Out of GOs	7.2359
*yieI*	b3716	Predicted IM protein	Out of GOs	6.6363
*gcvB*	b4443	GcvB small regulatory RNA	Regulatory	7.4984
*yeeN*	b1983	Conserved protein	Regulatory	5.3142
*rihA*	b4142	GroES, chaperone binds to Hsp60 in pres. Mg-ATP, suppressing its ATPase activity	Stress response	5.9806
*cyoE*	b0428	Heme O synthase	Structural molecule activity	5.9183
*cysC*	B2750	Adenylylsulfate kinase	Sulfur metabolism	7.0141
*cysH*	b2762	3′-Phospho-adenylylsulfate reductase	Sulfur metabolism	11.2772
*cirA*	b2155	OM receptor involved in uptake of ferric dihyroxybenzoylserine	Transport	9.4700
*fecA*	b4291	Ferric citrate OMP FecA	Transport	19.9369
*fecB*	b4290	Ferric dicitrate ABC transporter - periplasmic binding protein	Transport	14.5864
*fruB*	b2169	Fructose PTS permease - FruB subunit	Transport	5.8528
*glpT*	b2240	GlpT glycerol-3-P MFS transporter	Transport	8.6190
*lamB*	b4036	Phage lambda receptor protein; maltose high-affinity receptor	Transport	18.0411
*livK*	b3458	Leucine ABC transporter - periplasmic binding protein	Transport	6.0726
*malE*	b4034	Maltose ABC transporter - periplasmic binding protein	Transport	174.0512
*malF*	b4033	Maltose ABC transporter - membrane subunit	Transport	19.1719
*malK*	b4035	Maltose ABC transporter - ATP binding subunit	Transport	14.8026
*nmpC*	b0553	OMP protein; locus of qsr prophage	Transport	11.7561
*proX*	b2679	Glycine betaine/proline ABC transporter - periplasmic binding protein	Transport	7.3363
*sbp*	b3917	Sulfate/thiosulfate ABC transporter - periplasmic binding protein Sbp	Transport	11.2712
*tnaB*	b3709	TnaB tryptophan ArAAP transporter	Transport	9.9672
*tsx*	b0411	Nucleoside channel; receptor of phage T6 and colicin K	Transport	6.2757

^a^Retrieved from EcoCyc database, ^b^Biological functions were assigned according to the EcoCyc database for *E. coli* strain MG1655 and broadly grouped on the basis of GO terms [17].

**Table 4:** Average expression data of upregulated genes during EXP/STA2 comparison based on resultant FDR value from the RP analysis equal or less to 0.05 in compared growth phases during SA production cultures

**Gene**	**Locus**	**Protein name**^**a**^	**Cellular function GO**^**b**^	**Average expression**
*ykfE*	b0220	(*ivy*) Protein: inhibitor of vertebrate C-type lysozyme	Amino acid metabolism	29.2978
*cysK*	b2414	Enzyme: cysteine synthase A	Amino acid metabolism	83.0796
*gcvH*	b2904	Polypeptide: glycine cleavage system H protein	Amino acid metabolism	30.4955
*gcvP*	b2903	Enzyme: glycine decarboxylase	Amino acid metabolism	33.3045
*gcvT*	b2905	Enzyme: aminomethyltransferase	Amino acid metabolism	38.3509
*gdhA*	b1761	Enzyme: glutamate dehydrogenase	Amino acid metabolism	20.9785
*glyA*	b2551	Enzyme: serine hydroxymethyltransferase	Amino acid metabolism	23.7380
*tnaA*	_b3708	L-cysteine desulfhydrase/tryptophanase	Amino acid metabolism	139.6125
*tnaL*	b3707	*tna* operon leader peptide	Amino acid metabolism	62.9786
*ompT*	b0565	Enzyme: OM protease VII (OM protein 3b)	Catalytic activity	35.7201
*fimC*	b4316	Polypeptide: periplasmic chaperone, required for type 1 fimbriae	Cell envelope	63.3469
*fimD*	b4317	Polypeptide: OM protein; export and assembly of type 1 fimbriae	Cell envelope	25.6637
*nlpA*	b3661	Polypeptide: lipoprotein-28	Cell envelope	48.3194
*ydiQ*	b1697	Polypeptide: putative subunit of YdiQ-YdiR flavoprotein	Electron carrier activity	29.9950
*nrdA*	b2234	Protein: ribonucleoside diphosphate reductase 1, α subunit dimer	Nucleotide/nucleoside metabolism	37.6063
*yciW*	b1287	Polypeptide: predicted oxidoreductasePolypeptide: predicted oxidoreductase	Out of GOs	116.1438
*yieI*	b3716	(*cbrB*) Polypeptide: predicted IM protein	Out of GOs	31.9090
*yeeN*	b1983	Polypeptide: conserved protein	Regulatory	22.0828
*yhiE*	b3512	(*gadE*) Polypeptide: GadE DNA-binding transcriptional activator	Regulatory	46.2077
*yhiW*	b3515	(*gadW*) Polypeptide: GadW DNA-binding transcriptional dual regulator	Regulatory	37.7545
*hdeA*	b3510	Protein: HdeA dimer, inactive form of acid-resistance protein	Stress response	32.4174
*hdeB*	b3509	Polypeptide: acid stress chaperone	Stress response	56.2185
*b0023*	b0023	Polypeptide: 30S ribosomal subunit protein S20	Structural molecule activity	45.8728
*cysC*	b2750	Enzyme: adenylylsulfate kinase	Sulfur metabolism	52.8463
*cysD*	cysD	Polypeptide: CysD	Sulfur metabolism	236.2573
*cysH*	b2762	Enzyme: 3′-phospho-adenylylsulfate reductase	Sulfur metabolism	194.5029
*cysJ*	b2764	Enzyme: sulfite reductase, flavoprotein subunit complex	Sulfur metabolism	62.3545
*ydjN*	b1729	Polypeptide: predicted transporter	Transport	43.4944
*dppB*	b3543	Polypeptide: dipeptide ABC transporter - putative membrane subunit	Transport	23.0830
*dppF*	b3540	Polypeptide: dipeptide ABC transporter - putative ABC binding subunit	Transport	32.6107
*fecA*	b4291	Polypeptide: ferric citrate OMP FecA	Transport	23.6130
*malE*	b4034	Maltose ABC transporter - periplasmic binding protein	Transport	230.4651
*ompC*	b2215	Transporter: OMP C	Transport	61.2832
*oppB*	b1244	Polypeptide: murein tripeptide ABC transporter/peptide ABC transporter - putative membrane subunit	Transport	44.3550
*proV*	b2677	Polypeptide: glycine betaine/proline ABC transporter - ATP binding subunit	Transport	34.7488
*proW*	b2678	Polypeptide: glycine betaine/proline ABC transporter - membrane subunitPolypeptide: glycine betaine/proline ABC transporter - membrane subunit	Transport	35.6683
*proX*	b2679	Polypeptide: glycine betaine/proline ABC transporter - periplasmic binding protein	Transport	48.2957

^a^Retrieved from EcoCyc database, ^b^Biological functions were assigned according to the EcoCyc database for *E. coli* strain MG1655 and broadly grouped on the basis of GO terms [17].

**Table 5:** Average expression data of upregulated genes during STA1/STA2 comparison based on resultant FDR value from the RP analysis equal or less to 0.05 in compared growth phases during SA production cultures

**Gene**	**Locus**	**Protein name**^**a**^	**Cellular function GO**^**b**^	**Average expression**
*argA*	b2818	Acetylglutamate synthase	Amino acid metabolism	87.9837
*argB*	b3959	N-acetylglutamate kinase	Amino acid metabolism	45.8143
*argC*	b3958	N-acetylglutamylphosphate reductase	Amino acid metabolism	53.4932
*argD*	b3359	N-succinyldiaminopimelate-aminotransferase/acetylornithine transaminase	Amino acid metabolism	21.9053
*argE*	b3957	Acetylornithine deacetylase	Amino acid metabolism	18.7485
*argG*	b3172	Argininosuccinate synthase	Amino acid metabolism	65.2192
*argH*	b3960	Argininosuccinate lyase	Amino acid metabolism	23.7859
*argI*	b4254	Ornithine carbamoyltransferase chain I	Amino acid metabolism	33.2672
*carA*	b0032	Polypeptide: CarA	Amino acid metabolism	107.7785
*carB*	b0033	Polypeptide: CarB	Amino acid metabolism	23.9781
*ilvG_1*	b3767	Acetolactate synthase II, large subunit, N-ter fragment	Amino acid metabolism	75.4038
*ilvG_2*	b3768	Acetolactate synthase II, large subunit, N-ter fragment	Amino acid metabolism	24.6648
*ilvM*	b3769	Polypeptide: IlvM	Amino acid metabolism	16.9332
*metE*	b3829	Cobalamin-independent homocysteine transmethylase	Amino acid metabolism	59.0097
*serA*	b2913	α-Ketoglutarate reductase/D-3-phosphoglycerate dehydrogenase	Amino acid metabolism	15.4266
*ybaS*	b0485	Glutaminase	Amino acid metabolism	27.4637
*fimC*	b4316	Periplasmic chaperone, required for type 1 fimbriae	Cell envelope	20.5382
*ydiQ*	b1697	Putative subunit of YdiQ-YdiR flavoprotein	Electron carrier activity	32.4583
*ydiR*	b1698	Putative subunit of YdiQ-YdiR flavoprotein	Electron carrier activity	32.3715
*ydiT*	b1700	Putative ferredoxin	Electron carrier activity	28.0548
*arnB*	b2253	UDP-L-Ara4O C-4′ transaminase	Lipid metabolism	20.7697
*arnC*	b2254	Undecaprenyl phosphate-L-Ara4FN transferase	Lipid metabolism	33.1058
*yhiD*	b3508	Predicted Mg(2+) transport ATPase	Lipid metabolism	52.2858
*narY*	b1467	Nitrate reductase Z, β subunit	Other metabolic process	18.7104
*narZ*	b1468	Nitrate reductase Z, α subunit	Other metabolic process	13.4094
*yciE*	b1257	Conserved protein	Other metabolic process	18.9020
*ydiS*	b1699	Putative flavoprotein	Other metabolic process	20.0646
*ygdI*	b2809	Putative lipoprotein	Out of OGs	15.8477
*slp*	b3506	Starvation lipoprotein	Out of OGs	16.3895
*ybaY*	b0453	Predicted OM lipoprotein	Out of OGs	31.4852
*yhjR*	b3535	Conserved protein	Out of OGs	17.5298
*yhiE*	b3512	GadE DNA-binding transcriptional activator	Regulatory	224.6027
*yhiF*	b3507	Polypeptide: predicted DNA-binding transcriptional regulator	Regulatory	33.7016
*yhiW*	b3515	GadW DNA-binding transcriptional dual regulator	Regulatory	33.4367
*ykfE*	b0220	Inhibitor of vertebrate C-type lysozyme	Regulatory	40.8144
*gadB*	b1493	Glutamate decarboxylase B	Stress response	94.5462
*hdeA*	b3510	HdeA dimer, inactive form of acid-resistance protein	Stress response	125.3695
*hdeB*	b3509	Acid stress chaperone	Stress response	104.9129
*hdeD*	b3511	Acid-resistance membrane protein	Stress response	140.6337
*katE*	b1732	Heme d synthase/hydroperoxidase	Stress response	15.6533
*cysD*	b2752	Polypeptide: CysD	Sulfur metabolism	34.5193
*cysH*	b2762	3′-Phospho-adenylylsulfate reductase	Sulfur metabolism	15.6801
*hisP*	b2306	Lysine/arginine/ornithine ABC transporter/histidine ABC transporter - ATP binding subunit	Transport	17.8485
*potG*	b0855	Putrescine ABC transporter - ATP binding subunit	Transport	18.0562
*artJ*	b0860	Arginine ABC transporter - periplasmic binding protein	Transport	120.3908
*gadC*	b1492	Glutamic acid: 4-aminobutyrate antiporter	Transport	28.9528
*hisM*	b2307	Lysine/arginine/ornithine ABC transporter/histidine ABC transporter - membrane subunit	Transport	44.5442
*narU*	b1469	NarU MFS nitrate/nitrite antiporter	Transport	62.1767
*ompC*	b2215	OMP C	Transport	37.5373
*yggB*	b2924	Mechano sensitive channel MscS	Transport	22.3863

^a^Retrieved from EcoCyc database, ^b^Biological functions were assigned according to the EcoCyc database for *E. coli* strain MG1655 and broadly grouped on the basis of GO terms [17].

## Discussion

### Upregulation of genes involved in sugar transport

Genes encoding sugar transporters that were found to be upregulated in the EXP/STA1 comparison included the sugar porin LamB (*lamB*), the periplasmic binding protein, the membrane subunit and the ATP binding subunit components of the ABC maltose transport system (*malE and malFK*, respectively) [17] and the GlpT glycerol 3-P MFS transporter (*glpT*), whereas in the EXP/STA2 comparison, the upregulation of the non-specific OMP *ompC* (OmpC), a general porin and the periplasmic binding protein of the maltose ABC transporter (*malE*) was observed. A previous transcriptomic analysis of our group determined by RT-qPCR in cultures of the parental strain PB12 in M9 minimal broth showed the upregulation of the *galP* gene, suggesting that GalP protein is the main transporter used for glucose import in this PTS^−^ strain [18], as is the case in a derived PB12 strain for L-Phe production grown in M9 broth supplemented with 5 g/L of YE [19]. However, our GTA showed that in the SA-producing strain PB12.SA22, the *galP* gene was not significantly upregulated during EXP growth and the STA phase. This result was also observed in GTA for the parental strain PB12 in the same culture conditions (data not shown), suggesting that in both strains grown in complex fermentation broth for SA production, the OMP OmpC, LamB, and the maltose ABC transporter, have an important role in the transport of glucose because previous works reported the participation of these proteins in the uptake of glucose in *E. coli*[20-23]. All these genes were found to be upregulated in the EXP and STA1 phases, associated with the consumption of >30% of the initially added glucose to the fermentation broth; however, only in the STA1/STA2 comparison was detected the upregulation of *ompC*. Differential expression of these transporters is in agreement with the absence of glucose in the supernatant culture observed at the middle STA phase (26 h). Interestingly, the gene encoding the glycerol-3-P (G3P) MFS transporter (GlpT) (*glpT*) was also detected upregulated during the EXP/STA1 comparison but not during STA1/STA2. G3P plays a major role in glycolysis and phospholipid biosynthesis in *E. coli*. G3P is transported by GlpT and is reduced by aerobic or anaerobic G3P dehydrogenase into dihydroxyacetone phosphate, which is converted into fructose-1,6-diphosphate or glyceraldehyde-3-phosphate and then enters the glycolysis pathway. In phospholipid biosynthesis, G3P forms the backbone of all phospholipid molecules and the polar groups of phosphatidylglycerol and cardiolipin [24]. Upregulation of this gene in strain PB12.SA22 is intriguing because G3P was not present during the SA production cultures. GlpT could be involved in phospholipid biosynthesis during the EXP/STA1 comparison because it was previously reported, and indirect evidence suggests that GlpT expression may be regulated by a byproduct from the glycerolipid biosynthetic pathway [24].

### Upregulation of genes involved in the amino acid transport and metabolism

During the EXP/STA1 comparison, *livK*, encoding a subunit of the ABC transporter for leucine, and *tnaB* (part of the *tnaCAB* operon) encoding the TnaB tryptophan ArAAP transporter, which is proposed to be involved in tryptophan scavenging [25], were upregulated. The EXP/STA2 comparison revealed the upregulation of the genes for the putative membrane protein and the ATP binding components of the BppBCDF dipeptide ABC transporter (*dppB* and *dppF*), which is associated with the transport of proline-glycine as source of proline, histidine-glutamic acid, and leucine-tryptophan [26,27]; the putative membrane protein of the murein tripeptide ABC transport system (*oppB*), which can transport oligopeptides of up to five residues in length as well as recycle cell-wall peptides, but it has been reported to lack affinity for free amino acids [28-31]; and the complete glycine betaine/proline ABC transporter encoded by the *proVWX* operon [17]. Expression of this operon was reported to substantially increase at high osmolarity to scavenge glycine, betaine, proline, taurine, ectoine, carnitine as the precursor for betaine, and choline to achieve high intracellular concentrations of these osmoprotectants [32-34]. During the STA1/STA2 comparison, the periplasmic binding protein of the arginine ABC transporter (*artJ*); the 4-aminobutyrate antiporter glutamic acid GadC (*gadC*), a member of the APC superfamily of amino acid transporters [35]; and the integral membrane subunit and the ATP-binding component of the lysine/arginine/ornithine ABC transporter and the histidine ABC transporter (*hisM and hisP*, respectively) were found to be upregulated [17].

Differential upregulation of those genes encoding peptide and amino acid transporters during the entire fermentation process suggests that during batch culture for SA production, strain PB12.SA22 imports the substrates that are available in the fermentation broth into the cell because they are supplied by YE, which contains 6% amino nitrogen, supplying peptides and 18 of the 20 proteinogenic amino acids (with the exception of asparagine and glutamine) [36]; however, differential upregulation of amino acid transporters observed during the EXP, STA1 and STA2 phases suggests a possible differential amino acid requirement by the cell during the fermentation process, particularly for leucine and tryptophan during the EXP/STA1 comparison and leucine, arginine, glutamic acid, lysine and histidine during the STA1/STA2 comparison. Amino acid availability in the fermentation broth provided by YE almost certainly alleviates the cellular requirements because YE contains all these amino acids [36]. Finally, in the EXP/STA1 and EXP/STA2 comparisons, *ompT*, encoding protease VII was upregulated; this protein is capable of cleaving several peptides at the center of paired basic residues but not at single basic residues, suggesting a distinct mechanism for trypsin-like proteases [17]. Although OmpT is not involved in transport, it is possibly related to peptide assimilation in strain PB12.SA22 during SA production.

Together with the upregulation of genes encoding peptide and amino acid transporters, several genes encoding enzymes involved in amino acid catabolic or biosynthetic pathways were differentially upregulated during the EXP growth and STA phases. Among biosynthetic genes encoding the enzymes involved in the complete cysteine biosynthesis from sulfate, CysD, CysC, CysH (*cysD, cysC* and *cysH*, respectively) [17] were found upregulated during the EXP/STA2 and STA1/STA2 comparisons, while CysJ (*cisJ*), encoding sulfite reductase (NADPH), which catalyzes the 6-electron reduction of sulfite to sulfide, one of several activities necessary for the biosynthesis of cysteine from sulfate [17], was found to be upregulated only in the EXP/STA1 comparison. During the EXP/STA2 comparison, we detected the upregulation of those genes encoding hydroxymethyltransferase (*glyA*) involved in the conversion of serine to glycine; the enzyme cysteine synthase A (*cysK*) catalyzing the conversion of O-acetyl-L-serine to L-cysteine [37-39] (although, in the absence of a sulfur source, this enzyme catalyzes the slow conversion of O-acetyl-L-serine into pyruvate + acetate + ammonia, or the conversion of O-acetyl-L-serine into simple serine [40]; and glutamate dehydrogenase (*gdhA*) involved in the NADPH-dependent amination of α-ketoglutarate to yield L-glutamate. Catabolic genes upregulated during the EXP/STA1 and EXP/STA2 comparisons included *tnaCA* (part of the *tnaCAB* operon), which encodes the tryptophanase leader peptide and the tryptophanase involved in tryptophan degradation to indole + PYR + ammonia + H^+^. During the EXP/STA1 and EXP/STA2 comparisons, the entire glycine cleavage system (GCV) (*gcvTHP* operon), a multienzyme complex catalyzing the reversible oxidation of glycine, yielding carbon dioxide, ammonia, 5,10-methylenetetrahydrofolate and a reduced pyridine nucleotide [17,41] was also upregulated. Tetrahydrofolate serves as a recipient for C_1_ units generated during glycine cleavage to form the methylene group, which are of central physiological importance, as will be discussed below [41-43].

Another group of upregulated genes found during the EXP/STA1 comparison, were the genes involved in ammonia dependent conversion of aspartate to asparagine (amino acid not supplied by YE), by asparagine synthetase (*asnA*) and the catabolic genes involved in the conversion of aspartate to Fum + ammonia + H^+^ by aspartate ammonia-lyase (*aspA*), which is the first step in the proline degradation pathway to glutamic acid by proline dehydrogenase (*putA*), and in serine degradation to PYR + ammonia + H^+^ by L-serine deaminase II (*sdaB*) [17]. Notably, a group of upregulated genes during the STA1/STA2 comparison include genes encoding the entire arginine biosynthesis pathway and the ornithine biosynthesis pathway (*argA, argCBH, argG, argD* and *argI*) and carbamoyl phosphate synthetase (*carAB*), which catalyzes the first committed step in the biosynthetic pathways for the production of arginine and pyrimidine nucleotides. The small subunit of this enzyme (*carA*) also hydrolyzes glutamine to glutamate and ammonia [44]. *ilvG*_1 and *ilvG*_2, part of the *ilvLXG_1G_2MEDA* operon [45], are pseudogenes encoding subunits of acetohydroxybutanoate synthase / acetolactate synthase, an essential enzyme that catalyzes the biosynthesis of α-aceto-α-hydroxybutyrate for the isoleucine pathway and of α-acetolactate for the valine biosynthesis when *E. coli* K-12 was grown on acetate or oleate as the sole carbon source [46]. Other genes upregulated in the STA1/STA2 comparison encode cobalamin-independent homocysteine transmethylase (*metE*), which catalyzes the final step of *de novo* methionine biosynthesis in the absence of exogenously supplied vitamin B12 (cobalamin); α-ketoglutarate reductase/D-3-phosphoglycerate dehydrogenase (*serA*), which catalyzes the first committed step in the biosynthesis of L-serine from 3-P-glycerate; and glutaminase (*ybaS*), which catalyzes the degradation of glutamine to yield glutamate [17].

The main cellular role of amino acids is as structural blocks for the synthesis of proteins. Their availability determines the growth capabilities of cells, thereby defining the extent of the EXP growth phase, particularly for auxotrophic strains. Because strain PB12.SA22 is auxotrophic for aromatic amino acids due to the deletion of the *aroK* and *aroL* genes, its growth depends of the extracellular availability of these essential amino acids present in YE. As it was observed in Figure 2B, when tryptophan was depleted from the fermentation broth, this strain decreased its *μ* even in the presence of <70% residual glucose, showing that the availability of this amino acid is a determinant factor for growth capabilities of strain PB12.SA22 after 9 h of fermentation, although the strain could use additional (non-specified) carbohydrates supplied by YE (17.5%) [36] or other amino acids as possible carbon sources.

**Figure 3:**
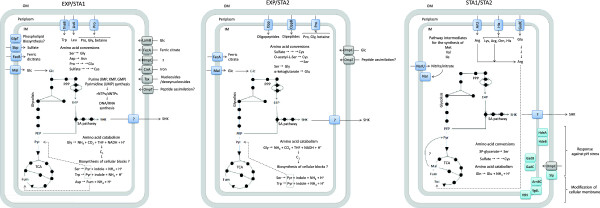
**Proposed metabolic and physiologic traits of *E. coli *PB12.SA22.based on relevant upregulated genes during EXP and STA GTA.** OM, IM, Glycolysis, PPP, TCA and SA pathway are illustrated schematically. Arrows indicate the direction of transport or reaction; dotted arrows indicate suggested or unknown mechanisms.

Based on our GTA data, differential upregulation of genes encoding glycine transport components (operon *proVWX*) and the genes involved in the synthesis of serine from 3-P-glycerate and its subsequent transformation to glycine by upregulation of *glyA* and *serA*, no differential expression of *serB* and *serC* genes (Additional file 1) and the upregulation encoding genes for the GCV suggest an important role for these two amino acids in strain PB12.SA22 because they are most likely used additionally as protein components to yield C_1_ units, which provide precursors for diverse important cellular process; indeed, it was reported that during growth on glucose, *E. coli* employs ~ 15% of the carbon assimilated in serine and its metabolites, such as the synthesis of cysteine, phospholipids, and glycine, whereas glycine is used in the synthesis of purines and heme-containing compounds. C_1_ units derived from serine and glycine are used in the synthesis of purines, histidine, thymine, pantothenate, and methionine and in the formylation of the aminoacylated initiator fMet-tRNA used to start translation in *E. coli.* In turn, these compounds are involved in other essential cellular process demanding the supply of C_1_ units [41,42], suggesting that particularly in the EXP/STA1 and EXP/STA2 comparisons C_1_ units derived from serine and glycine could be channeled for the biosynthesis of diverse cellular components used mainly for cellular maintenance of strain PB12.SA22 (Figure 3).

Interestingly, the genes encoding the complete biosynthetic pathway for arginine from glutamate ArgA, ArgB, ArgC, ArgD, ArgE, ArtI, ArgG and ArgH (*argA, argB, argC, argD, argE, argF, argG* and *argH*, respectively) and from bicarbonate to carbamoyl-phosphate (*carAB*) [17] were found to be upregulated in the STA1/STA2 comparison, whereas they were downregulated during the EXP phase (Additional file 1), suggesting an important role of this amino acid during the STA phase distinct from its role as a protein component. Arginine is also available in the supernatant broth because it is also supplied by YE (3.03%) [36]. This amino acid is transported by the arginine ABC transporter ArtPMQJI (*argP, argM, argQ, argJ* and *argI*, respectively); however, among these genes, *artI* encodes the periplasmic binding component and is the unique found upregulated gene of this transporter, whereas the remaining genes were found without changes in their expression, suggesting that in addition to the upregulation of genes encoding the complete biosynthetic pathway for arginine from glutamate and bicarbonate, this amino acid is also transported from the fermentation broth during the STA phase. Arginine can be degraded by two pathways, the arginine succinyltransferase (AST) pathway yielding succinate, which enters to TCA, and the arginine decarboxylase/agmatinase pathway, which also yields succinate. This last pathway yields the intermediate putrescin, which connects arginine degradation with the biosynthetic pathways of the polyamines putrescine, spermidine, cadaverine and aminopropylcadaverine [17]. Interestingly, unlike *E. coli* K-12, wild-type *E. coli* strains are unable to use L-arginine as a carbon source, but they can use this amino acid as a nitrogen source [47,48]. Based on this evidence, our transcriptomic data suggest that arginine could be used by strain PB12.SA22 as a source of succinate to recycle C through TCA or as a source for polyamines during the STA phase for SA production. However, data mining of the RP method analysis for microarray data comparison between STA1/STA2 showed that none of the genes of the arginine AST degradation (operon *astCADBE*) and arginine decarboxylase/agmatinase (*adiA, speB, speF, puuAP, puuDRCBE* and *sad*) pathways were significantly upregulated (Additional file 1). Polyamines are reported as necessary for cell growth because they are major polycations in cells, together with Ca^2+^ and Mg^2+^. Polyamines and Mg^2+^, which are present in higher free concentrations than Ca^2+^, can bind to intracellular polyanions such as nucleic acids and ATP to modulate their function [49]. Polyamines have also been associated with stabilizing membranes and stimulating several enzymes [17], and furthermore, spermidine was found to donate a portion of its molecule for the enzymatic biosynthesis of hypusine, a unique amino acid that plays a crucial role in cell proliferation [50]. Because strain PB12.SA22 ceased EXP growth by hour 9 of cultivation and entered a pseudo STA phase until the end of the fermentation, it is unlikely that arginine could be used as a polyamine precursor, suggesting that it could be used to supply succinate to TCA, through the gluconeogenic capabilities developed by strain PB12 [18,51], to make PEP available for use in SA production, particularly during the late STA phase in the absence of glucose (Figure 3).

### Possible associations between amino acid transport, catabolism and pH stress response

Amino acids such as aspartate, serine and tryptophan could be used potentially by *E. coli* as carbon sources under certain growth conditions. Catabolism of aspartate by aspartate ammonia-lyase, AspA (*aspA*) yields Fum + ammonia + H^+^; catabolism of L-serine by serine deaminase, SdaB (*sdaB*) yields PYR + ammonia + H^+^; whereas the catabolism of tryptophan catalyzed by tryptophanase (*tnaA*) yields PYR + ammonia + indole + H^+^. In turn, fumarate and PYR could fuel TCA or increase intracellular PYR availability, respectively [17]. However, amino acid deamination is a metabolic process displayed by *E. coli* to contend with alkaline stress that supplies carbon for bacterial growth under this condition, particularly when growing in complex peptide-rich media. As cell density increases, extracellular pH alkalinizes and released ammonia is deprotonated and volatilized, whereas the C skeleton is channeled into acids [52-54]. Based on our transcriptomic data showing the upregulation of genes such as *tnaA*, *aspA* and *sdaB* during the EXP and STA phases, we propose that intracellular availability of amino acids such as aspartate, cysteine and tryptophan, either by importing them from the extracellular environment (as they are provided by YE) or by interconversion from other sources, e.g., cysteine from O-acetyl-L-serine by CysK (*cysK*) [17] (also upregulated during the EXP/STA2 comparison), have an important role in a possible cellular response of *E. coli* PB12.SA22 during SA production to extracellular alkalinization because upregulation of *tnaA*, *aspA* and *sdaB* suggests that their respective enzymes deaminate aspartate, cysteine and tryptophan to PYR or Fum. In addition to the upregulation of these genes, a previous report indicates that as a consequence of cellular exposure to neutral pH conditions, *E. coli* 3110 showed a higher expression of 93 genes (grouped as the neutral high expressed cluster showing highest expression at pH 7.0 and lower expression at both pH extremes), including *fecA, fecB, fecE, lamB, livK, malE, malF* and *malK*[55]. Interestingly, all these genes were found to be upregulated in strain PB12.SA22 in the EXP/STA1 comparison, with exception of *malE*, which was also upregulated in the STA1/STA2 comparison.

Several genes encoding proteins involved in the AR in *E. coli* were found to be upregulated during the EXP and STA phases, including the chaperone-based AR proteins HdeA and HdeB, which belong to the *hdeAB* acid stress operon involved in the periplasmic acid stress response that prevent periplasmic-protein aggregation at acidic pH; the IM protein HdeD (*hdeD*), which is required for acid resistance [56], was found to be upregulated during the STA1/STA2 comparison, and GadB and GadC (*gadBC* operon) were upregulated in the STA1/STA2 phases and are components of the Gad system (the glutamate decarboxylase system), the major AR system in *E. coli* under extreme acidic conditions [17]. GadB (together with GadA) are pyridoxal 5′-phosphate (PLP)-dependent enzymes that convert glutamate to γ-amino butyric acid (GABA) and carbon dioxide in a reaction that consumes a cytoplasmic proton. GABA is transported out of the cell by the IM antiporter GadC in exchange for more extracellular glutamate [57], whereas GadW is a transcriptional dual regulator (*yhiW* or *gadW*) [17,57,58]. Several regulatory genes of the Gad system (*gadA, gadE, gadW and gadX*), as well as the chaperone-related genes *hdeABD*, were placed in a unique cluster termed the acid fitness island [59], but interestingly, more genes in this island were found to participate in acid resistance, including the OM lipoprotein Slp (*slp*) and the transcriptional regulator YhiF (*yhiF*) (both genes were upregulated in the STA1/STA2 comparison in our transcriptomic study), which are required to protect cells against excreted toxic metabolites, including the accumulated anions of dissociated weak acids after growth at low pH such as lactate, succinate, and formate [57]. Additionally, GadE serves as the global transcriptional activator for many genes involved in stress response, glutamante biosynthesis, and in the biosynthesis of membrane components. Auto-induction of GadE requires the alternative sigma^S^ factor responsible for the transcription of many genes in the STA phase and another unidentified factor, whereas P2 and P3 of *gadE* are activated by GadX (or YhiX) and GadW (or YhiW) during the STA phase [57].

The simultaneous upregulation of genes encoding proteins involved in acid pH stress response with the upregulation of catabolic amino acid pathway to revert an apparent alkalinization has been reported to have a common connection, the protein GadB [52]. As was discussed above, high extracellular pH induces transcription of several genes encoding enzymes generating ammonia, such as TnaA, CysK, SdaB and AsnA, by deaminating tryptophan, serine, cysteine and aspartate, respectively. Expression of *gadB* is controlled by sigma^S^ during the STA phase in minimal growth medium; however, induction of this gene has been observed during growth in complex medium between pH 5.5 and 8.0 [52,55,60]. Induction of the RpoS dependent- oxidative AR system has been reported due to the presence of glutamate in YE. Glutamate and glutamine appear to activate a preformed RpoS-dependent system that is produced by entry into the STA phase [60]. According to this observation, simultaneous upregulation of the AR Gad system associated with the induction of genes involved in alkaline pH stress observed during SA production could be induced by the presence of glutamate in YE, which is transported by GadC. Additionally, upregulation of *hdeAB* during the EXP/STA2 comparison can be explained by the inductor effect of glutamate because these genes are also activated by GadE and GadW [17].

It has been established that the OM envelope and the periplasmic space are cellular compartments exposed essentially to extracellular pH [52]. In bacterial strains used for metabolite production such as PB12.SA22, during SA production, it is not surprising that the periplasmic side of the IM, the periplasmic space and the inner side of the OM could be exposed to low acid stress because SA is exported from the cytoplasm and then diffuses into the extracellular environment; conversely, the external side of the OM could be exposed to low alkaline stress as a consequence of the addition of NH_4_OH to maintain fermentor pH ≅ 7.0. This supposition makes sense given the observed upregulation of genes involved in acid pH stress response, simultaneous with the upregulation of the deaminating amino acid pathway to reverse an apparent alkalinization during SA production by strain PB12.SA22 (Figure 3).

### Upregulation of genes involved in iron and sulfur transport and its metabolism

Iron and sulfur seems to play an important physiological role during SA acid production by strain PB12.SA22 because some genes encoding transporters for these ions were found to be upregulated. Among these, *fecBE* was found to be upregulated in the EXP/STA1 comparison, and *fecA* was observed in the EXP/STA2 comparison. The *fecABC* operon genes are located in the *fecABCDE* operon and encode the ferric citrate OMP FecA (*fecA*), the IM ferric dicitrate ABC transporter (*fecB*), and the ATP binding component (*fecE*) [61]. Additionally, it was found to be upregulated in the EXP/STA1 comparison the OM receptor Cir (*cirA*), a TonB dependent iron-siderophore complex involved in iron uptake and regulated by both cellular iron content and growth [62]. Regarding sulfur, the upregulation of the periplasmic binding protein (Sbp) sulfate/thiosulfate ANC transporter (*sbp*) in the EXP/STA1 comparison was observed, which is associated with the transport both of sulfate and thiosulfate that are used as sulfur sources [63]. Finally, in the STA1/STA2 comparison, we also found that the genes *narU*, *narZ* and *narY* (part of the *narUZYWV* operon) were upregulated, which encode the nitrite/nitrate transporter (*narU*) and the *σ* and *β* subunits of the IM located nitrite/nitrate reductase (*narZ* and *narY*, respectively).

Iron is essential for the growth of *E. coli*, as is emphasized by the variety of processes in which iron-containing proteins take part, including their structural association to proteins involved in electron transport or playing important roles, particularly for iron-sulfur proteins involved in amino acid and pyrimidine biosynthesis (glutamate synthase, dihydroorotate dehydrogenase), and the TCA (aconitase, succinate dehydrogenase), as well as in electron transport (ferredoxin) and non-heme, non-iron-sulfur proteins required for DNA synthesis (ribonucleotide reductase), protection from superoxide radicals (superoxide dismutase), and interestingly, aromatic amino acid biosynthesis (DAHP synthase) [64]. Exogenous ferric citrate (supplied in the fermentation broth for SA production as ammonium iron (III) citrate), is transported across the OM by FecA, and a signal is transmitted across the OM to the IM protein FecR, which transmits the signal across the IM, thereby activating (through the sigma^70^ factor) the cytoplasmic family protein FecI, which directs RNA polymerase to express the *fecABCDE* operon [17,61,65].

### Upregulation of genes involved in nucleotide/nucleoside transport and biosynthesis

During the EXP/STA1 comparison, upregulation was observed for the gene encoding OMP Tsx (*tsx*), a protein that has been proposed to function *in vivo* as a pore that specifically facilitates the permeation of nucleosides and deoxynucleosides across the OM due to its specificity for free nucleobases or monophosphate nucleosides [22,66]. Regarding nucleotide/nucleoside metabolism, were found upregulated genes involved in pyrimidine ribonucleoside salvage, *rihA* and *upp* encoding ribonucleoside hydrolase 1 (pyrimidine-specific) and uracil phosphoribosyltransferase, respectively, which catalyze the sequential conversion of uridine → uracil → UMP [17]. Regarding pyrimidine ribonucleotide *de novo* biosynthesis, was found upregulated the gene *pyrB* encoding the catalytic subunit of the aspartate carbamoyltransferase, an enzyme catalyzing the conversion of L-asparte + carbamoyl-P to N-carbamoyl-L-aspartate in reactions involved in the biosynthesis of UMP in this pathway [17]. Finally, regarding the adenine and adenosine salvage pathway, upregulation was detected for the *gpt* gene*,* encoding xanthine-guanine phosphoribosyltransferase, which catalyzes the conversion of hypoxanthine to IMP, a common precursor for the guanosine and adenosine nucleotides in the *de novo* biosynthetic pathway [17].

Xanthine-guanine phosphoribosyltransferase is also involved in the xanthine and xanthosine salvage pathway, catalyzing the conversion of xanthine to XMP, which is then channeled to the purine nucleotide *de novo* biosynthesis pathway, and also catalyzes the transformation of guanine to GMP in the guanine and guanosine salvage pathway. GMP is, in turn, channeled to the guanosine nucleotide *de novo* biosynthesis pathway [17]. Additionally, upregulation was observed for *guaC*, encoding the GMP reductase, which catalyzes the conversion of GMP to IMP by a reductive deamination, and the *nrdA* gene, encoding the *α*-subunit dimer of the ribonucleoside diphosphate reductase 1 enzyme, which catalyzes the conversion of nucleotides to deoxynucleotides, an essential step during DNA synthesis, including its role in the chromosome replication and repair processes [17]. Data mining of the microarray expression comparison between the EXP/STA1 phases showed no significant expression differences based on the FDR value ≤ 0.05 for other genes involved in salvage pathway of pyrimidine ribonucleotides (*pyrH, ndk, pyrG, cmk*) or for the pyrimidine *de novo* biosynthesis pathway (*pyrC, pyrD, pyrE, pyrF, ndk or pyrG*) (Additional file 1). Interestingly, *nrdA* was found to be upregulated in the late STA phase, but no other genes involved in the purine and pyrimidine pathways discussed above were found to be significantly upregulated in the STA1/STA2 comparison.

All genes encoding enzymes related to the so-called nucleotide salvage pathways are involved in transformations of purine nucleotides derived from exogenous sources, whereas in the *de novo* pathways, they are synthesized from simpler precursors [67]. Our transcriptomic data, showed the upregulation of few genes encoding enzymes catalyzing reactions both in the *de novo* and salvage pathways involved in the synthesis of the purines IMP, XMP, GMP and the pyrimidine nucleotide UMP. Upregulation of the above described genes ensures the availability of rNTPs and dNTPs for RNA and DNA synthesis, respectively, from both purine (IMP, XMP, GMP) and pyrimidine (UMP) essential nucleotides during the growth stage of strain PB12.SA22 (Figure 3).

### Modification of the cellular membrane during the STA phase

In the STA1/STA2 comparison, important differences were observed in the upregulation of several genes possibly involved in inner and OM structure modification and the response to diverse environmental processes with respect to the EXP/STA1 comparison. These include the *arnBC* genes encoding an undecaprenyl transferase involved in the modification of lipid A phosphates with 4-amino-4-deoxy-L-arabinose (L-Ara4N), which modifies the lipid composition of the outer face of the periplasmic membrane [68]; *yciE*, encoding YciE, a conserved protein of unknown function, which has been observed to be induced under osmotic stress imposed by NaCl in both aerobic and anaerobic conditions [69]; *ygdI,* encoding an IM putative lipoprotein [17]; *slp*, encoding a STA phase lipoprotein (Slp) that has been proposed to take part in acid resistance because its expression was observed when cells were grown in pH 5.5 to 4.5 under conditions known to induce glutamate dependent acid resistance compared to pH 7.4, and also associated with YhiF to protect the cell against toxic metabolites [54]. Expression of *slp* was previously observed to increase 3-5-fold in the STA carbon starvation and was found to form homo-oligomer complexes tethered to the OM [70]. Other genes found to be upregulated were *ydhI* (or *yhhE*), a putative ATPase transporter involved in high cell dependent acid resistance [71]; and the *ompC* gene*,* encoding OmpC. Although the possible role of OmpC in glucose transport during EXP growth was discussed above, expression of *ompC* and OmpC protein level have been demonstrated to also be influenced by a wide variety of environmental conditions including pH, osmolarity, temperature, concentration of certain toxins, and growth phase [72]. OmpC and OmpF are reported as the major constituents of the OM in *E. coli*, accounting for approximately 2% of the total protein content of the cell [73]. The role of the OMPs in the modification of the OM has been previously reported, indicating that the OmpC/OmpT:OmpA ratio increased in total membrane protein analysis of an evolved *E. coli* resistance to increasing isobutanol concentrations correlating with upregulation of these genes, resulting in changes in the OM structure, accomplished with modification in membrane composition and peptidoglycan structure [74]. Interestingly, in support of our proposition regarding the possible role of those genes discussed in this section, particularly during the STA phase, cell resistance to lysis during the total RNA extraction procedure showed that PB12.SA22 modified its cellular surface properties because the protocol originally developed for successful total RNA extraction in the mid EXP phase (5 h) was modified for the extraction in the STA phases, thereby enhancing the cellular lysis step to yield a high amount of total RNA suitable for microarray analysis.

### Sigma factor regulatory networks controlling the expression profiles in the EXP and STA phases

**Figure 4:**
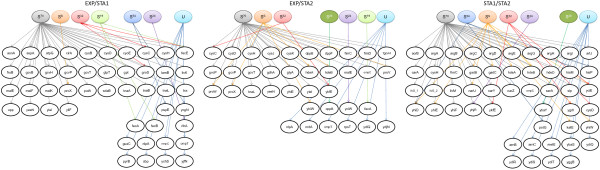
**Proposed transcriptional regulatory network of the sigma factors in *E. coli *PB12.SA22 in EXP/STA1, EXP/STA2 and STA1/STA2 comparisons.** U, unspecified sigma factor.

We constructed each regulatory network of the sigma factors controlling upregulated genes observed in EXP/STA1, EXP/STA2 and STA1/STA2 comparisons. The sigma regulatory interactions were retrieved from the comparisons between the information stored in the Ecocyc and Regulon DB databases [17,45]. The comparisons show that the sigma^70^ (RpoD) was the main sigma factor targeting promoters for genes encoding amino acid metabolism, ATP biosynthetic/catabolic process, transport, electron carrier activity, nucleotide/nucleoside metabolism, cell envelope and stress response (Figure 4). However, although RpoD is the primary sigma factor during EXP growth, targeting a wide range of promoters that are essential for normal growth [75] as is shown in Figure 4, transcription of several upregulated genes were possibly controlled by sigma^70^ and at least one or two more sigma factors. However, the transcription of other sets of genes are possibly controlled only by alternative sigma factors, and interestingly, 17%, 16% and 18% of significantly upregulated genes during the EXP/STA1, EXP/STA2 and STA1/STA2 comparisons, respectively, are controlled by unspecified sigma factors.

Alternative sigma factors possibly controlling transcription of several genes simultaneously to sigma^70^ during EXP/STA1 comparison are sigma^S^, sigma^32^ and sigma^19^. Interestingly, the *fecA, fecB* and *fecE* genes (encoding the ferric citrate OMP, the periplasmic binding protein and the ATP binding subunit of the dicitrate ABC transporter, respectively), are located in an operon with a promoter targeted by sigma^19^ controlling the expression of the *fecABCD* operon genes, whereas the expression of the contiguous gene in the operon, *fecE*, is controlled by sigma^70^ and sigma^19^, thus suggesting, as discussed above, an important role for iron during EXP growth of strain PB12.SA22. During EXP/STA2 comparison, sigma^S^ and sigma^70^ were found to be controlling the expression of six genes involved in transport (membrane subunit of the glycine betaine/proline ABC transporter encoded in the operon *proVWX*), acid stress response (*hdeAB* operon) and DNA binding transcriptional activators (*yhiE, yhiW* genes), respectively [17]. Data retrieved from the EcoCyc and Regulon DB databases suggested that during the STA phase (the STA1/STA2 comparison), sigma^70^ was most likely associated with the transcription of 35 upregulated genes; however, in this comparison, it was proposed that the sigma factors ^54^, ^S^, ^32^ and ^24^ possibly simultaneously participated with sigma^70^ RpoD in the co-transcription of 12 genes, including those involved in acid stress response (*gadBC, hdeAB* and *yhiD*), nitrite/nitrate transport (*yciE*), osmotic stress response (*yciE, katE*) and DNA binding transcriptional activators (*yhiE, yhiW* genes) [17] (Figure 4).

Sigma^70^ accounts for 60-95% of the total pool of cellular sigma factors during normal EXP growth [17]. Changes to typical growth conditions, such as heat shock, acid stress or growth into the STA even in rich broth, lead to the replacement of RpoD with other sigma factors such as RpoS, which is considered to be the master regulator of the general stress response in *E. coli.* RpoS is practically absent in rapidly growing cells but is strongly induced during entry into the STA phase and/or many other stress conditions and is essential for the expression of multiple stress resistances [76]. When wild-type *E. coli* is grown in glucose-limiting conditions and, interestingly, in PTS^−^ strains such as PB12.SA22 parental strain PB12, transcription of several central metabolism and especially glycolytic genes turn on under the control of RpoS, which has been proposed as a second vegetative sigma factor with major impact not only on stress tolerance but on the entire cell physiology under non-optimal growth conditions [77,78]. However, the comparison of our transcriptomic data showed that the significantly upregulated genes across EXP/STA1, EXP/STA2 and STA1/STA2 that are controlled by RpoD average 71%, and interestingly, in the comparison between EXP/STA2 and STA1/STA2, the sigma^S^ factor was involved in the simultaneous upregulation of 22% of those genes controlled by sigma^70^.

Data mining of differentially expressed genes during all performed comparisons showed that all genes encoding sigma factors were not significantly up- or downregulated based on the FDR value ≤ 0.05 (Additional file 1). This result suggests a relevant role for those positive or dual transcriptional factors targeting transcription initiation sites of upregulated genes.

### Concluding remarks

During batch cultures for SA production using complex fermentation broth (including 25 g/L of glucose and 15 g/L of YE), strain PB12.SA22 ceased EXP growth even in the presence of a high amount of residual glucose, indicating that growth was not associated with glucose consumption, depending to some extent of the availability of nutrients supplied by YE. This hypothesis was supported by the observation that EXP growth cessation is associated with the total consumption of available tryptophan in the supernatant, entering a STA-like phase where a brief increment in biomass was observed. During this stage, cells consumed residual glucose in the supernatant, suggesting that biomass produced during the EXP phase entered a resting cell-like condition producing SA. Because no changes were observed in the regulation of genes involved in CCM and the SA pathway between the EXP and STA phases, it is possible to propose that this strain transports and catabolizes extracellular glucose mainly for SA production in the STA phase until 26 h of cultivation, where glucose was completely consumed. Those metabolic capabilities regarding glucose catabolism and synthesis of precursors PEP and E4P for SA production could be a consequence of the permanent scavenging condition proposed previously for parental strain PB12, as a consequence of the inactivation of the PTS operon and diverse genetic changes developed during an evolutive adaptation process from which the PB12 strain was obtained [18,79].

GTA of *E. coli* strain PB12.SA22 during SA production between the mid EXP phase (5 h), the early STA phase (9 h) and the late STA phase (44 h) showed no significant differences in absolute expression in genes encoding enzymes of CCM, glycolysis, the PPP, TCA, the glyoxilate shunt, acetate metabolism and gluconeogenic enzymes, such as those in the SA pathway. However, important differences were observed in the upregulation of genes encoding proteins involved in sugar, amino acid, nucleotide/nucleoside and iron and sulfur transport; metabolic processes, particularly amino acid catabolism and biosynthesis; nucleotide/nucleoside salvage; acid stress response; and the modification of the cell membrane in the EXP/STA1, EXP/STA2 and STA1/STA2 comparisons. Figure 3, shows a proposed metabolic model based on relevant upregulated genes observed during comparisons in strain PB12.SA22 during SA production in batch-fermentor cultures grown in a complex broth. During the EXP/STA1 comparison, strain PB12.SA22 has an important transport activity through OMPs and IM proteins that possibly transport glucose, sulfur, iron, amino acids, peptides and nucleotide/nucleosides whereas amino acid interconversion and catabolism process were maintained. In the EXP/STA2 comparison, an important reduction in transport activity was observed particularly for some amino acids and possibly peptides supplied by the YE, while important amino acid interconversion and catabolism processes, such as nucleotide/nucleoside biosynthesis, were maintained. In both comparisons, it is important to highlight the catabolism of amino acids such as serine, tryptophan and asparagine (EXP/STA1) and serine and tryptophan (EXP/STA2), which results in PYR or Fum, and particularly the possible catabolism of glycine through the GCV, suggesting the possibility that PYR and Fum could be channeled to the CCM, whereas the catabolism of cysteine could result in the availability of C_1_ units for the biosynthesis of cellular blocks. Finally, during the STA1/STA2 comparison, only the transport of arginine, glutamic acid, and nitrite/nitrate and the biosynthesis of arginine from glutamic acid were observed. The availability of arginine and its possible catabolism could yield succinate, which could be channeled to the TCA. As was discussed, glucose is completely consumed at 26 h of cultivation, but SA accumulation continued until the end of fermentation. The results suggest how strain PB12.SA22 continues producing SA during the late STA phase. Even if the catabolism of arginine could fuel carbon into TCA and, possibly, the gluconeogenic capabilities of this strain could supply PYR and PEP, the availability of E4P becomes a limiting resource for DAPH synthesis and the subsequent accumulation of SA. Another important group of genes upregulated in the STA1/STA2 comparison are possibly involved in the cellular response to pH stress genes (*gadB, gadC, hdeA, hdeB*) and several genes encoding proteins possibly involved in both outer and IM modifications as a response to environmental conditions imposed on the cell at the end of the fermentation.

Finally, the network reconstruction based on different sigma factors controlling the expression of upregulated genes of each condition showed that even sigma^S^ has been proposed to be the master regulator of the STA, and sigma^70^ also plays an important role in controlling some upregulated genes with no alternative reported sigma factor or any sharing its regulatory region with a sigma^S^ promoter or an alternative sigma factor. This sharing is an important property of regulatory networks, which gives them plasticity to adapt to different environmental conditions.

## Methods

### Bacterial strain and growth conditions

The SA-producing strain PB12.SA22 (PTS^−^ Δ*aroK* Δ*aroL* pTOPO*aroBaroE,* pJLB*aroG*^fbr^*tktA*) [10] was shake-flask cultured in 125 mL baffled flasks containing 10 mL of Luria-Bertani broth supplemented with 30 *μ*g/mL of kanamycin, 15 *μ*g/mL of gentamycin, 20 *μ*g/mL of chloramphenicol, and 30 *μ*g/mL of tetracycline. Cultures were incubated overnight in a shaker (New Brunswick Scientific, Edison, USA) at 37°C, 300 rpm. An aliquot of 150 *μ*L of this culture was used to inoculate a 250 mL baffled flask with 50 mL of fermentation medium containing 25 g/L of glucose and 15 g/L of YE [8,10]. Biomass concentration was determined, and calculations were performed to adjust inoculum size at OD_600 nm_ = 0.35 and to inoculate batch cultures (by triplicate) in an Applikon autoclavable glass Bio Reactor one L fermentor with a 500 mL working volume of fermentation medium supplemented with required antibiotics. Fermentor vessels were connected to an Applikon ADI 1010 BioController and ADI 1025 controllers to monitor temperature, pH, impeller speed and DO. Batch fermentations were run for 50 h at 37°C, pH 7.0 (maintained by addition of 3.0% of NH_4_OH). Impeller speed of no less than 500 rpm was used to maintain DO levels at 20% air saturation [80]. Gene-carrying plasmids were induced by adding 0.1 mM of isopropyl *β*-D-1-thiogalactopyranoside.

### Analytical procedures

Biomass concentrations were monitored each hour during the first eight h of cultivation. After this point, concentrations were monitored every six h until the end of the fermentation (50 h). Triplicated samples (1.5 mL) were withdrawn from each reactor and OD_600 nm_ was determined spectrophotometrically (Beckman DUR-70 Spectrophotometer). Supernatant samples for the determination of SA, DHP, DHS, and GA were prepared by centrifuging one mL of fermented broth at 12,000 rpm for 1 min. To remove any residual cell in the supernatant, the centrifuged samples were filtered through 0.45 *μ*M nylon membranes. SA, DHS, and GA concentrations were determined by HPLC as described previously [10], whereas DAHP concentrations were determined by the thiobarbituric acid assay [81]. Because this method does not distinguish between DAHP and DAH, in this work, DAHP levels corresponded to the sum of both compounds [82]. Glucose concentration was assessed by a biochemical analyzer (YSI 2700 Select). Aromatic amino acids phenylalanine, tyrosine and tryptophan, present in culture supernatants, were quantified using a Phenomenex Synergy Hydro RP18 column (150 by 4.6 mm; 4 *μ*m) attached to an Agilent 1100 HPLC system (Agilent Technologies). Running conditions were as follows: mobile phase, 0.2% of trifluoroacetic acid 40% of methanol; flow, 0.5 mL/min. Detection was performed by photodiode array at 280_nm_[82].

The *μ* and the *qs* were calculated during the EXP growth phase. *qs* was calculated as the differential change in S, with time (t) normalized to the biomass concentration ( $\text{qs}=\frac{\mu }{\text{Yx}/s}$). A predetermined correlation factor (1 OD_600 nm_ corresponded to 0.37 g/L of DW) was used to transform OD_600 nm_ values into cell concentrations for *qs* and *μ* calculation.

TACY determinations were based on the combined molar yields of DAHP, DHS, SA, and GA [10]. Results were reported as the average of triplicated samples with their associated standard deviation.

### RNA extraction procedures

One aliquot of 5 mL from each of three fermentor cultures performed were collected at the middle of the EXP growth phase (5 h, DO_600 nm_ = 7.00), the early STA state (9 h, DO_600 nm_ = 13.00) and the late STA state (44 h, DO_600 nm_ =15.2) (resulting in triplicate samples for each time), and transferred to a 15-mL Falcon tube containing 500 μL of RNA later solution (Ambion). Total RNA extraction was performed using a modification of the previously reported hot phenol-based procedure. Previous methodology was used successfully for total RNA extraction from cultures of *E. coli* JM101, and its PTS^−^-derivative PB11 and PB12 strains, grown in batch-fermentor cultures in minimal M9 medium and collected at DO_600 nm_ = 1.0 [18]; however, direct application of this methodology to biomass collected from cultures of strain PB12.SA22 for SA production in a complex medium resulted in poor total RNA yield, particularly in samples collected during STA. To obtain total RNA of high quality (non-degraded and with high absorbance DO_260/280_ and DO_230/260_ ratios) and concentration required to perform successful GTA, the original RNA extraction procedure was modified and optimized. The integrity of extracted total RNA was evaluated by agarose gel electrophoresis, and quality was evaluated by determination of concentration and the DO_260/280_ and DO_260/230_ ratios in a Nanodrop-2000c spectrophotometer (Thermo Scientific). Samples were adjusted to a final concentration of total RNA = 1 *μ*g/*μ*L. Aliquots of 20 *μ*L of total RNA were shipped to the Precision Biomarker company for microarray experiments (http://www.precisionbiomarker.com).

### Microarray design, experiments and data analysis

Total RNA extracted from each samples withdrawn at 5, 9 and 44 h from triplicated fermentor cultures, were entirely processed by Precision Biomarker. Sample preparation and processing included cDNA synthesis, cDNA fragmentation, and the preparation of the hybridization mixture, hybridization with the Affymetrix GeneChip® *E. coli* Genome 2.0 (Affymetrix), washing, staining, and scanning of the microarrays, which were all performed according to the manufacturers’ procedures. Raw data acquisition and gene expression data analysis were performed by Precision Biomarker using background correction, normalization and summarization. The resulting databases corresponding to the analyzed samples were analyzed in our laboratory by retrieving expression data from the array of *E. coli* MG1655, including in the Affymetrix GeneChip® *E. coli* Genome 2.0. Average data of triplicates from each growth phase sampled were used to calculate the relative expression value between EXP relative to STA1 (EXP/STA1 (2^^(EXP-STA1)^), STA1 relative to STA2 (STA1/STA2 (2^^(STA1-STA2)^) and EXP relative to STA2 (2^^(EXP-STA2)^). The resulting databases were formatted using a Perl language program from http://www.gla.ac.uk/schools/computing/?CFID=21255484&CFTOKEN=17693561 for analysis by the rank product method to identify differentially expressed (upregulated or downregulated) genes [15,16].

## Abbreviations

μ: Specific growth rate; AR: Acid response; C1: One-carbon units; CCM: Central carbon metabolism; CHA: Chorismic acid; DAHP: 3-Deoxy-D-*arabino*heptulosonate 7-phosphate; DAHQ: 3-Dehydroquinic acid; DHQ: Dehydratase 3-Dehydroquinate dehydratase; DHQ: Synthase 3-Dehydroquinate synthase; DHS: 3-Dehydroshikimic acid; DO: Dissolved oxygen; DW: Dry weight; E4P: Erythrose 4-phosphate; EPSP: 5-Enolpyruvyl-shikimate-3-phosphate; EXP: Exponential growth phase; fbr: Feedback resistant; FDR: False discovery rate; Fum: Fumarate; GA: Gallic acid; GMP: Guanosine-monophosphate; GO: Gene ontology; GTA: Global transcriptomic analysis; IM: Inner or cellular membrane; IMP: Inosine-5′-phosphate; OAA: Oxaloacetate; OD: Optical density; OM: Outer membrane; OMP: Outer membrane porin; OSP: Oseltamivir phosphate; PEP: Phosphoenol pyruvate; PPP: Pentose-phosphate pathway; PTS: Phosphoenolpyruvate:carbohydrate phosphotransferase system; qS: Glucose consumption rate; RP: Rank products; S3P: Shikimate-3-phosphate; SA: Shikimic acid; STA: Stationary phase; TACY: Total aromatic compounds yield; TCA: Tricarboxylic acid cycle; UMP: Uridine-5′-phosphate; XMP: Xanthosine-5′-phosphate; YE: Yeast extract.

## Competing interests

The authors declare that they have no competing interests.

## Authors’ contributions

LCT, RMG, FB and AE participated in the design of this study. LCT and LMM developed the total RNA extraction methodology. RA was involved in fermentations. LCT and RMG analysed the microarray data analysis. AE, FB, RMG, GG and LCT participated in the analysis of the results, as well as in writing and critical review of the manuscript. All authors have read and approved the manuscript.

## Supplementary Material

Additional file 1 Differentially upregulated and downregulated genes during EXP/STA1, EXP/STA2 and STA1/STA2 comparisons based on the application of the RP defined by a FDR value ≤ 0.05. Click here for file
